# Jean Dubousset 1936–2025

**DOI:** 10.2340/17453674.2025.44138

**Published:** 2025-06-25

**Authors:** Acke Ohlin, Ilkka J Helenius, Tamas S Illes, Paul Gerdhem

**Affiliations:** Lund University, Malmö, Sweden; Department of Orthopaedics and Traumatology, University of Helsinki and Helsinki University Hospital, Helsinki, Finland; Department of Orthopedics & Traumatology, Brugmann University Hospital, Université Libre de Bruxelles/Vrije Universiteit Brussel, Brussels, Belgium; Department of Surgical Sciences, Uppsala University, Uppsala, Sweden, email: paul.gerdhem@uu.se

**Figure:**
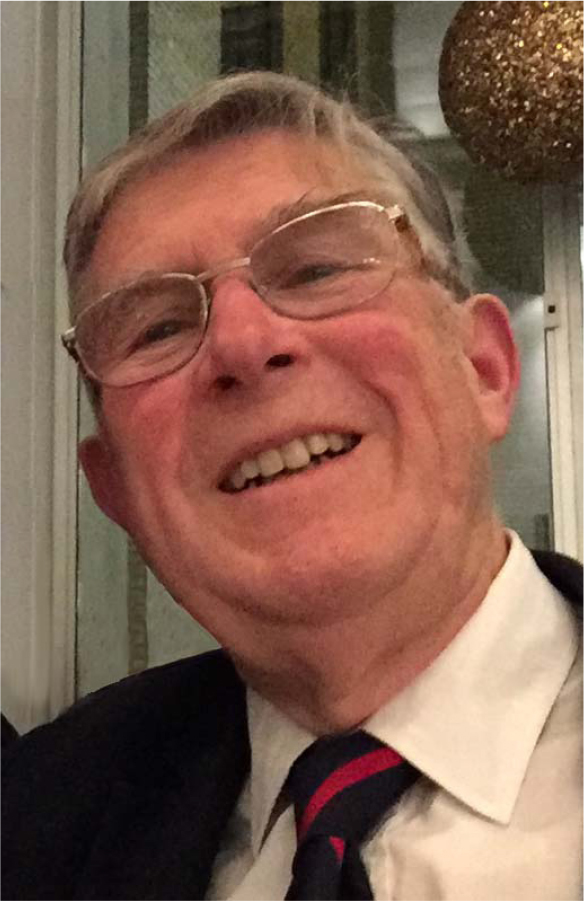
Jean Dubousset

Jean Dubousset, a member of the Académie Nationale de Médecine, former professor at René Descartes University – Paris, and one of the world’s most influential and innovative spine surgeons, recently passed away in his 89th year.

As colleagues who had the privilege of meeting Professor Dubousset, and some of us also being his students, we would like to pay tribute to his memory by highlighting a few of his outstanding observations on the treatment and surgical care of spinal deformities.

Throughout his career, Professor Dubousset attached great importance to the spatial analysis of spinal deformities, with particular attention to the sagittal and axial appearance of scoliosis. In his opinion, the origin of scoliosis development is to be sought in changes in the axial plane of the spine.

Together with Yves Cotrel, he developed the multisegmental Cotrel–Dubousset (CD) instrument for the spatial correction and fixation of spinal curvatures, which spread rapidly worldwide after its introduction in 1983. During one of his early CD surgeries, he observed with his insightful mind that rotating a rod bent according to the required physiological curvature resulted in significant deformity correction in all 3 planes of space. This technique, which he described as the “derotational maneuver,” has been used by almost every spinal deformity surgeon in the world during scoliosis correction surgeries.

He introduced the concept of the “cone of economy,” which describes the optimal alignment of the trunk. The cone of economy indicates a spatial section within which the trunk or upper body can be kept in balance with minimal energy, even with possible residual curvatures.

He also played a pioneering role in developing the non-fusion surgical treatment of “early onset scoliosis.” By developing the magnetic field-induced rod distraction concept, he significantly reduced the number of lengthening surgeries required to accompany spinal growth. Modern modifications of the technique are now widely accepted and used worldwide.

In collaboration with Nobel Prize-winning physicist Georges Charpak, he developed the EOS radiological imaging system to facilitate the analysis of spinal deformities. The system allowed for the reconstruction of the skeletal system as a whole in the standing position, with extremely low radiation exposure, based on simultaneous antero-posterior and lateral radiographs. The top-view display of the spatial reconstruction of the pelvis and spinal column opened up new perspectives in analyzing the axial appearance of spinal deformities.

Professor Dubousset loved teaching. He loved his students, too. He was always very open-minded and helpful to us, his younger colleagues. There was no clinical problem for which we turned to him in vain. He always had an adequate suggestion for a solution but, at the same time, he never forgot to praise us, which filled us all with enthusiasm for spine surgery again and again.

We remember him with deep respect and gratitude. Rest in peace, Jean.

**Acke Ohlin**, Lund University, Malmö, Sweden

**Ilkka J Helenius**, Department of Orthopaedics and Traumatology, University of Helsinki and Helsinki University Hospital, Helsinki, Finland

**Tamas S Illes**, Department of Orthopedics & Traumatology, Brugmann University Hospital, Université Libre de Bruxelles/Vrije Universiteit Brussel, Brussels, Belgium

**Paul Gerdhem**, Department of Surgical Sciences, Uppsala University, Uppsala, Sweden, email: paul.gerdhem@uu.se

